# Reviewed article: Alberti AM, Silva PBE, Azambuja FS, Godoy L, Concolatto FP, Lech GE, Stein AT. Epidemiological profile of surgical treatment of lung cancer in Brazil: retrospective analysis (2014-2023). Epidemiol Serv Saude. 2025:34;e20240427.

**DOI:** 10.1590/S2237-96222025v34e20240427.c

**Published:** 2025-08-04

**Authors:** Silvio Barberato-Filho

**Affiliations:** University of Sorocaba

**Table:** 

Rating	Excellent	Good	Average	Below average	Poor
Interest			✓		
Quality				✓	
Originality			✓		
Overall			✓		

**Table:** 

Questionnaire	Yes	No	Not applicable
Does the manuscript contain new and significant information to justify publication?	✓		
Does the Abstract (Summary) clearly and accurately describe the content of the article?		✓	
Is the problem significant and concisely stated?	✓		
Are the methods described comprehensively?		✓	
Are the interpretations and conclusions justified by the results?		✓	
Is adequate reference made to other work in the field?	✓		

## Manuscript Structure

Length of article is: Adequate

Number of tables is: Adequate

Number of figures is: Too many

Please state any conflict(s) of interest that you have in relation to the review of this paper (state “none” if this is not applicable): None

## Comments

Com o objetivo de contribuir para o aprimoramento do manuscrito, apresento as seguintes considerações/recomendações:

1. Título: incluir o período da análise, em anos.

2. O objetivo de propor estratégias para mitigação das disparidades regionais foi pouco desenvolvido, aparecendo apenas de forma discursiva no final da conclusão. Recomendo restringir o objetivo.

3. Adotar voz ativa (opte por ‘utilizaram-se ...’ em vez de “foram utilizadas para limpeza”).

4. Anglicismos, mesmo que usuais, devem ser evitados, optando por termo no vernáculo. Adotar itálico em palavras e expressões estrangeiras, quando não for possível substitui-las;.

5. Evitar uso indiscriminado de artigos indefinidos (um, uma...).

6. Garantir que informações da literatura estejam acompanhadas da respectiva citação e referência (exemplo, página 1 – linhas 59-60)

7. Explicitar no método o período de dez anos.

8. Hospitalização, internação e procedimento cirúrgico representam o mesmo dado? Uniformizar nomenclatura.

9. O que foi estudado sobre as consultas? Explicitar no método e citar no resumo.

10. Padronizar o número de casas decimais nos métodos, resultado e ilustrações: percentual 1 casa decimal, medida de associação: 2 casas decimais, p-valor: 3 casas decimais; apresentar p-valor exato com 3 casas decimais; ocorrências “0,000” devem ser grafadas como “<0,001”.

11. Não incluir espaço antes e após sinais (=, <, >, ≤, ≥ etc.). Medidas de dispersão devem ter seus intervalos separados por ponto e vírgula, precedidos da identificação da medida (ex.: IC95% 1,14; 2,23).

12. Informação sobre aprovação ética (ou dispensa) deve ser mencionada apenas na folha de rosto

13. Evitar repetição de resultados na discussão. Manter apenas o indispensável.

14. Evitar uso indiscriminado de maiúsculas.

15. Na Tabela 1 - Rever título (comparações múltiplas de...). Incluir rótulo na última coluna e/ou rever a forma de apresentação dos dados, agrupando colunas, se possível. Excluir coluna de significância estatística (manter apenas p-valor ajustado)

16. Figuras estão com erro nos títulos e na ordem.

17. Alterar textos internos das figuras 4a e 4b.

18. Avaliar se a informação da Figura 1 pode ser apresentada de outra forma no texto.

